# Extracellular Nucleotides Selectively Induce Migration of Chondrocytes and Expression of Type II Collagen

**DOI:** 10.3390/ijms21155227

**Published:** 2020-07-23

**Authors:** Marcin Szustak, Edyta Gendaszewska-Darmach

**Affiliations:** Institute of Molecular and Industrial Biotechnology, Faculty of Biotechnology and Food Sciences, Lodz University of Technology, Stefanowskiego 4/10, 90-924 Lodz, Poland; marcin.szustak@p.lodz.pl

**Keywords:** P2Y receptors, chondrocytes, migration, differentiation

## Abstract

The migration of chondrocytes from healthy to injured tissues is one of the most important challenges during cartilage repair. Additionally, maintenance of the chondrogenic phenotype remains another limitation, especially during monolayer culture in vitro. Using both the differentiated and undifferentiated chondrogenic ATDC5 cell line, we showed that extracellular nucleotides are able to increase the migration rate of chondrocytes without affecting their chondrogenic phenotype. We checked the potency of natural nucleotides (ATP, ADP, UTP, and UDP) as well as their stable phosphorothioate analogs, containing a sulfur atom in the place of one nonbridging oxygen atom in a phosphate group. We also detected *P2y1, P2y2*, *P2y4, P2y6*, *P2y12, P2y13*, and *P2y14* mRNA transcripts for nucleotide receptors, demonstrating that *P2y1* and *P2y13* are highly upregulated in differentiated ATDC5 cells. We showed that ADPβS, UDPβS, and ADP are the best stimulators of migration of differentiated chondrocytes. Additionally, ADP and ADPβS positively affected the expression of type II collagen, a structural component of the cartilage matrix.

## 1. Introduction

The cartilage is extremely susceptible to damage due to its load-bearing capability. If left untreated after injury, cartilage eventually degenerates, causing chronic osteoarthritis. The problem of cartilage repair is a growing civilization problem since self-regeneration of cartilage tissue is usually very limited. This is mainly due to the lack of blood vessels in this space, which are necessary to initiate the repair process. The problem is also the negligible amount of chondrogenic and progenitor cells surrounding the tissue area that migrate from the healthy tissue. The improved migration ability of chondrocytes is therefore considered to be essential for new therapeutic strategies, especially as in physiological settings, the dense pericellular matrix surrounding the chondrocytes makes migration a challenging process [1].

A number of studies have shown that different stimuli accelerate the rate of chondrocyte movement. For example, chondrocytes were demonstrated to be migrating in response to peripheral mononuclear blood-derived cells [2], insulin-like growth factor-1 [3], hyaluronic acid and basic fibroblast growth factor [4], sulfated hyaluronic acid [5], fibrin [6], or WNT5A protein [7]. We showed previously that extracellular nucleotides induce migration of HaCaT keratinocytes and HeLa cells via activation of P2Y nucleotide receptors [8,9]. Eight subtypes of P2Y family, namely P2Y1, P2Y2, P2Y4, P2Y6, P2Y11, P2Y12, P2Y13, and P2Y14, belong to the G protein-coupled receptors (GPCRs) family and regulate a variety of cellular processes, including proliferation, differentiation, secretion, cell adhesion, and migration. P2Y1, P2Y12, and P2Y13 are activated preferably by ADP while P2Y6 is activated by UDP. P2Y11 prefers ATP as an agonist while P2Y2 reacts to both ATP and UTP. UTP is also the P2Y4 agonist. Nucleotide sugar conjugate (UDP-glucose) activates the P2Y14 receptor [10]. However, there are scarce reports of the role of extracellular nucleotides on chondrogenic biology. Since some subtypes of P2Y receptors have been demonstrated to be expressed in chondrocytes [11], we hypothesized that purine and pyrimidine nucleotides may play relevant roles in the migration of chondrocytes. Expanding our understanding of purinergic signaling in the biology of chondrocytes could be translated in the future into clinical benefits. Unfortunately, extracellular nucleotides are hydrolyzed by specific ectonucleotidases present in both the cartilage matrix and synovial fluid [12]. Hence, hereby, we tested the ability to stimulate chondrocyte migration not only by unmodified nucleotides but also by their phosphorothioate analogs since our previous studies showed that replacing an oxygen atom with a sulfur in a phosphate group conferred significant resistance to enzymatic hydrolysis [9,13]. Stable analogs of nucleotides (e.g., ADPβS or UDPβS) are commonly utilized in studies addressing purinergic signaling [14,15].

Besides a low migratory ability, another limitation of adult chondrocytes is the phenomenon of dedifferentiation during their extensive expansion in vitro, the step required for autologous chondrocyte implantation (ACI) being the first-line cell therapy for critical cartilage defects [16]. Dedifferentiation of chondrocytes is accompanied with the gradual loss of cartilage-specific molecular markers, such as collagen type II (COL2A1), aggrecan, or SRY (sex-determining region Y)-box 9 (SOX9) transcription factor, whilst at the same time, the cells acquire a fibroblast-like phenotype, characterized by the expression of collagen type I (COL1A). Eventually, chondrocytes undergo final differentiation and become hypertrophic ones characterized by type X collagen and matrix metalloproteinase-13 expression. RUNX2 (Runt-related transcription factor 2) is responsible for hypertrophic differentiation and is directly inhibited by SOX9, a master transcription factor that regulates chondrocyte differentiation. Phenotypic conversion to hypertrophic chondrocytes is observed in the degenerative articular cartilage of osteoarthritis. Besides RUNX2, many genes are directly targeted by SOX9, including the gene encoding COL2A1 [17,18,19]. Therefore, we also evaluated if supplementation of the culture medium with extracellular nucleotides affects the expression of cartilage marker molecules.

As the first step of our studies, we analyzed the expression pattern of P2Y receptors in differentiated and undifferentiated chondrogenic ATDC5 cells. Subsequently, using both the differentiated and undifferentiated ATDC5 cell line, we compared the potential of various P2Y natural agonists and their stable analogs as agents affecting the migration and differentiation of chondrocytes.

## 2. Results

### 2.1. The Chondrogenic Phenotype of ATDC5 Growing Under Differentiating Conditions

**Figure 1:**
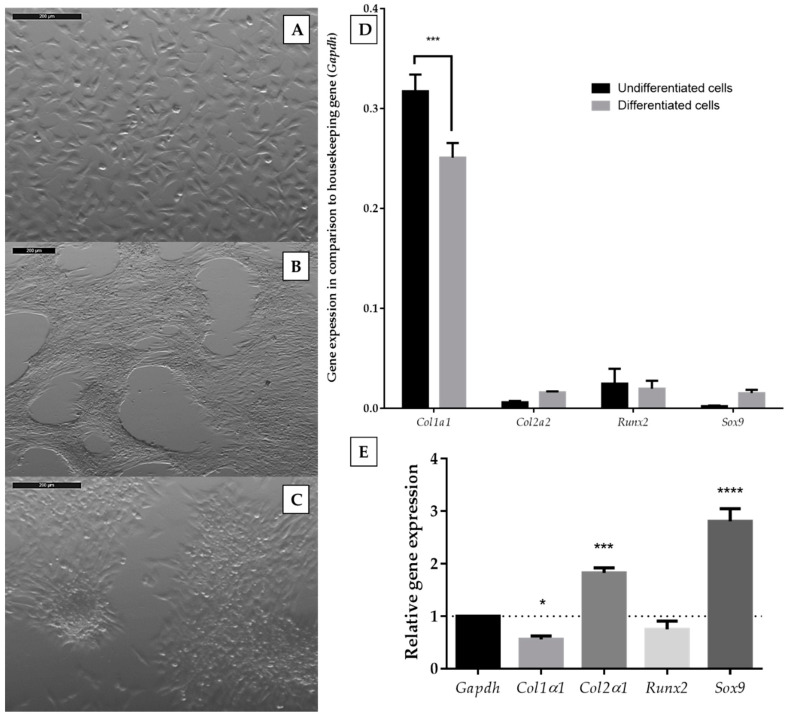
Morphology of ATDC5 cells grown for 14 days in standard growth medium (**A**) or in differentiation medium (**B**,**C**). The images were captured with a Leica 205C microscope and processed with the Leica Application Suite. The scale bar represents the length of 200 µm; expression levels of chondrocyte-specific genes in undifferentiated and differentiated ATDC5 cells in comparison to a *Gapdh* housekeeping gene (**D**), and relative gene expression calculated with Livak’s method [24] (**E**). Data represent the means ± standard error of the mean (SEM) from at least three independent experiments. Changes of genes expression between undifferentiated and differentiated cells for different genes were analyzed with two-way ANOVA with Tukey’s comparison test (D). Statistical difference between the control of a *Gapdh* housekeeping gene and tested genes were analyzed with one-way ANOVA (E). *p* < 0.05 (*), *p* < 0.001 (***), and *p* < 0.0001 (****) for gene expression significantly different from undifferentiated control cells.

The murine ATDC5 cell line is a well-recognized model for in vitro chondrogenesis studies. It retains the properties of chondroprogenitor cells and exhibits almost the same characteristics of chondrogenesis as mesenchymal stem cells [20,21]. Differentiation of ATDC5 cells can be induced by the presence of chondrogenic medium; however, literature reports define various compositions of such media. We chose differentiating medium consisting of DMEM/F12 (1:1), 2 mM glutamine, antibiotics (100 U/mL penicillin, 100 mg/mL neomycin, 2.5 µg/mL amphotericin B), insulin-transferrin-sodium selenite solution (ITS, 10 μg/mL insulin, 5.5 μg/mL transferrin, 67 ng/mL sodium selenite), 50 μg/ml L-ascorbic acid, 100 nM dexamethasone, and 10 ng/mL TGF-β2 [22] and confirmed its utility during the first stage of the study. Cells were grown in differentiation medium for 14 days because this period was described as necessary for sufficient differentiation [23]. As an undifferentiated control, we incubated ATDC5 cells in standard growth medium (DMEM/F12 (1:1), 2 mM glutamine, antibiotics, 5% fetal bovine serum). Then, we compared the morphological changes and expression of chondrogenic markers after the 14-day culture of ATDC5 in standard and differentiation medium. Cells growing in the monolayer culture in standard growth medium exhibited a fibroblast-like morphology (Figure 1A).

On the contrary, ATDC5 cells present in differentiating medium tended to group (Figure 1B) and form characteristic rounded clumps (Figure 1C). We also utilized reverse transcription quantitative polymerase chain reaction (RT-qPCR) for the quantification of type 1 collagen (*Col1α1),* type 2 collagen (*Col2α1)*, SRY-Box 9 (*Sox9)*, and runt-related transcription factor 2 (*Runx2)* mRNA expression. The expression of *Col1a1 was* downregulated in ATDC5 cells growing in a differentiating medium, while *Col2a1* and *Sox9* were upregulated (Figure 1D,E). The expression of *Runx2* did not change. Therefore, the chosen differentiating medium was used for further experiments.

### 2.2. Expression of P2Y Receptors in Differentiated and Undifferentiated ATDC5 Cells

**Figure 2:**
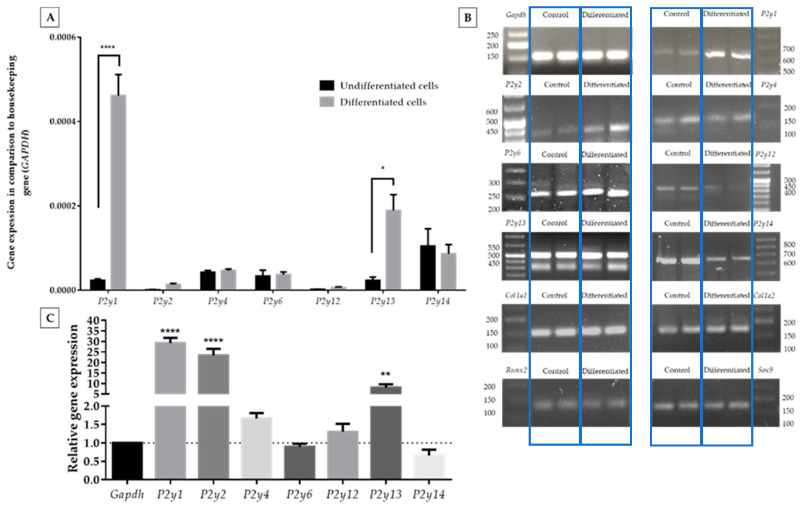
Comparison of P2Y receptor gene expression levels between ATDC5 cells grown in standard growth medium or in differentiation medium in comparison to a *Gapdh* housekeeping gene (**A**). Significant differences were calculated with two-way ANOVA with Tukey post-hoc test. The relative gene expression calculated with Livak’s method [24] showed changes in the expression level while *Gapgh* was constant (**C**). An image of DNA qPCR products separated on a 3% agarose gel (two repeated samples in each case) along with a DNA ladder and documented with the ChemiDoc Imaging system (**B**). qPCR products were separated with 3% agarose gel electrophoresis and documented with the ChemiDoc Imaging system (**B**). Statistical differences vs. control were calculated with one-way ANOVA. Data represent the means ± SEM from at least three independent experiments. *p* < 0.05 (*), *p* < 0.01 (**), and *p* < 0.0001 (****) for gene expression significantly different from undifferentiated control cells.

In the present study, we examined the expression of all P2Y receptors in ATDC5 cells using quantitative RT-PCR. P2Y11 expression was not studied due to the lack of a *P2y11* gene ortholog in the mouse genome [25]. Since the differentiation process may affect the expression level of P2Y receptors, at the beginning, we determined the expression pattern of P2Y receptors in undifferentiated and differentiated cells. In undifferentiated ATDC5 cells, *P2y4, P2y6*, and *P2y14* mRNA transcripts showed the highest expression of all receptors studied (Figure 2A).

The differentiation process highly upregulated the expression of *P2y1, P2y2,* and *P2y13* genes. The expression levels of all remaining receptors were upregulated (Figure 2A,C). The biggest increase was observed for the *P2y1* gene (ca. 30-fold) and *P2y2* gene (ca. 20-fold). However, the relatively high growth of *P2y2* was connected with its very low expression before the differentiation process. *P2y13* presented a ca.8-fold increase, which is confirmed by relative gene expression calculations. All qPCR products showed a desirable agarose gel pattern (Figure 2B).

### 2.3. Migration of ATDC5 Cells in the Presence of Extracellular Nucleotides

**Figure 3:**
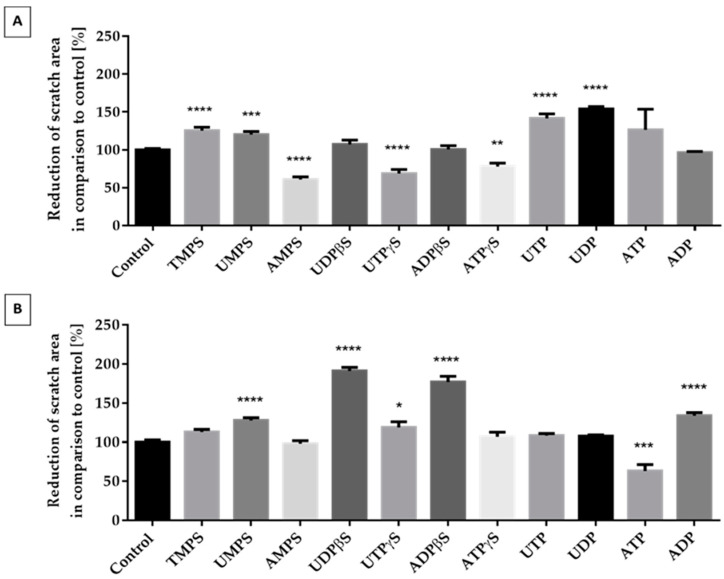
The effect of unmodified nucleotides and their phosphorothioate analogs on the migration of ATDC5 cells grown for 48 h in starving medium (**A**) or in differentiation medium (**B**). The extent of migration was documented with the Leica 205C microscope and processed in Leica Application Suite. Results represent a percentage of wound reduction in comparison to unstimulated control cells. Data represent the means ± SEM from at least six independent experiments. *p* < 0.01 (**), *p* < 0.001 (***), and *p* < 0.0001 (****) for reduction of scratch area significantly different from untreated control cells.

We targeted the evaluation of extracellular nucleotides as potentially promising stimulators of chondrocyte migration. We chose nucleoside 5′-diphosphates (ADP, UDP), nucleoside 5′-triphosphates (ATP, UTP), and nucleoside 5′-mono (TMPS, UMPS, AMPS), di (ADPβS, UDPβS), or triphosphorothioate analogs (ATPγS, UTPγS) for the studies. In the migration studies, we utilized serum-free starving medium instead of standard growth medium to suppress cell proliferation and deplete serum factors that could influence cell migration. We utilized a wound scratch assay, where a linear scratch was across the surface of a confluent plate dish. Then, confluent cells were stimulated with nucleotides for 48 h. Cells growing in starving medium were positively induced to migrate by natural nucleotides like UDP and UTP. Moreover, TMPS and UMPS also increased this process but to a lesser extent. We also observed an inhibitory effect in the case of AMPS, ATPγS, and UTPγS. All other tested compounds did not show any significant differences (Figure 3A). When differentiated cells were used, we noticed a dramatic change in the results (Figure 3B). In this case, only ATP diminished migration. ADP, UMPS, UTPγS, UDPβS, and ADPβS significantly accelerated cell migration. The most prominent activity was observed for ADPβS, UDPβS, and ADP.

### 2.4. The Influence of Extracellular Nucleotides on the Expression of *Cartilage*-Related *Gene Markers* in Differentiated and Undifferentiated ATDC5 Cells

**Figure 4:**
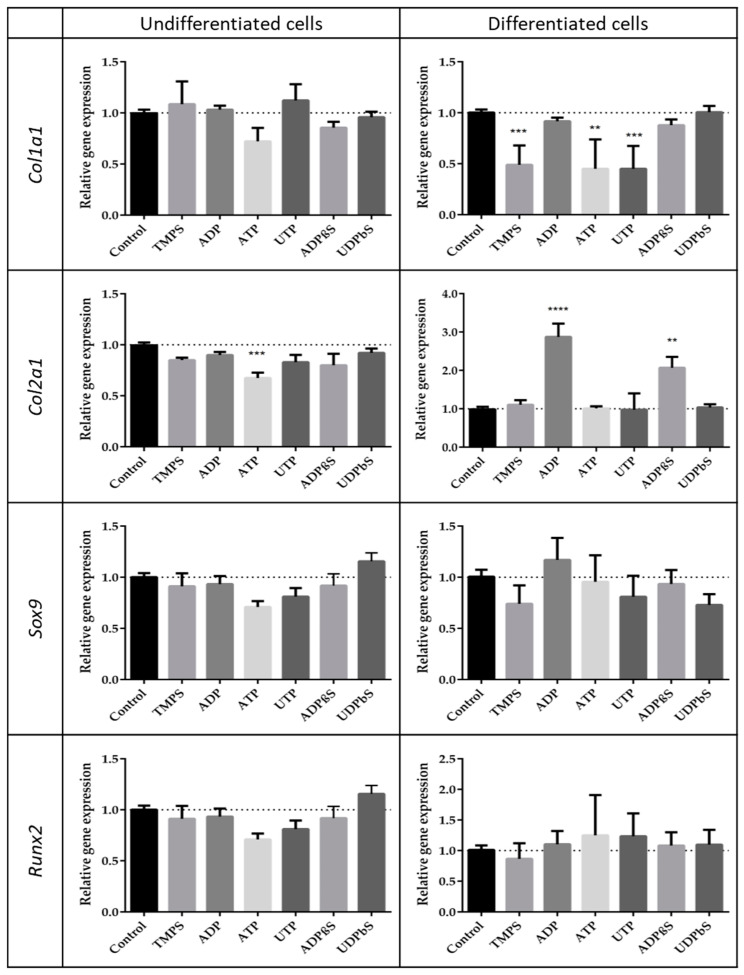
Expression patterns of chosen genes characteristic for the differentiation process in the presence of extracellular nucleotides or their phosphorothioate analogs. *Col1a1*, *Col2a1*, *Sox9,* and *Runx2* mRNA levels were measured by RT-qPCR in ATDC5 cells grown for 48 h in standard growth medium or in differentiation medium. Each value was normalized to *Gapdh* the same sample. Data represent the means ± SEM from at least six independent experiments. *p* < 0.01 (**) and *p* < 0.001 (***) for gene expression significantly different from untreated control cells.

Compounds that enhance the migration of chondrocytes cannot negatively affect the differentiation process. Hence, we analyzed the expression level of characteristic differentiation marker genes (*Col1a1*, *Col2a1*, *Sox9,* and *Runx2*) stimulated with selected nucleotides. We decided to test compounds with favorable (ADP, UDPβS, and ADPβS), neutral (UTP, TMPS), and negative (ATP) promigratory properties, taking into consideration differentiated ATDC5 cells. Among the nucleotides applied to undifferentiated cells, only ATP reduced the expression of all genes studied and the most significant downregulation was observed for *Col2a1*. More important results were obtained with nucleotides added to differentiated cells. Tested compounds did not significantly influence the expression level of the *Runx2* and *Sox9* genes. TMPS, ATP, and UTP significantly downregulated the *Col1α1* expression level. Interestingly, ADP and ADPβS strongly enhanced the expression characteristic for the differentiated cell gene *Col2α1* (Figure 4).

## 3. Discussion

The migration ability of chondrocytes is considered essential for the repair of cartilage since, in order to cure a defect, cells that fill the damaged tissue should migrate into such a space [2,7,26]. If left untreated after injury, cartilage degenerates and leads to chronic osteoarthritis (OA) [27]. Unfortunately, pathological changes that take place in OA include decreased cell migration that in turn can cause additional undesirable aggregation of chondrocytes and changes in matrix components [28]. The development of new agents targeting the migratory potential of chondrocytes is, therefore, a constant need for the development of new therapeutic strategies leading to the recovery of the original properties of cartilage. Chondrocyte migration enhancement therapy was also shown to positively affect tissue-engineered cartilage integration [29].

There is growing evidence to suggest that extracellular nucleotides play a significant role in the regulation of cartilage physiology [30]. Chondrocytes, for example, employ purinergic signaling as part of the mechanotransduction cascade, which promotes chemical signaling inside the cell. Increased expression of the P2Y2 receptor was indicated to be responsible for the elevated extracellular signal-regulated kinase (ERK1/2) phosphorylation in response to oscillatory fluid flow, which in turn regulates a variety of chondrocyte activities, including migration, proliferation, and differentiation [31].

We and others have shown previously that P2Y receptors are involved in nucleotide-induced migration of many cell types [8,9,32,33]. In this research, we showed that the supplementation of chondrocytes with extracellular nucleotides has a profound effect also on migration. In the resting state, the intracellular ATP concentrations are very high, ranging from 1 and 5 mM whereas it is much lower in the extracellular space, ranging in the nanomolar concentration (10–100 nM) [34]. However, the synovial fluid from normal and OA joints contains around 100 μM ATP [35]. Although it is difficult to measure the concentration of extracellular nucleotides within the extracellular space, extracellular UTP levels were demonstrated to account for 10–30% of the ATP levels in both resting and mechanically stimulated cultured cells [36]. Following mechanical stress, e.g., stretch and shear stress, in hypoxic conditions and during infection, local extracellular concentrations can be expected to ensure both P2Y receptors. The articular cartilage is subject and responsive to mechanical stimuli, and mechanical force on the extracellular matrix of the cartilage is transferred to the chondrocytes [37]. The migratory behavior was tested by a scratch assay, which was able to mimic a potential in vitro cartilage repair. Our studies demonstrate that migration of differentiated chondrocytes can be positively controlled by ADP, UMPS, UTPγS, UDPβS, and ADPβS; however, the most spectacular increase was observed for ADPβS, UDPβS, and ADP. Interestingly, in undifferentiated ATDC5 cells, we did not detect the promigratory activity of ADPβS, UDPβS, and ADP, implying that undifferentiated cells are less sensitive to their presence in the extracellular environment. In contrast, undifferentiated cells were positively triggered mostly by unmodified uridine nucleotides, suggesting the possibility that different signaling mechanisms may occur within these two cell populations. We hypothesize that the different responses between differentiated and undifferentiated ATDC5 cells may largely be due to different patterns of P2Y receptor expression.

Earlier studies have reported the expression of P2Y1, P2Y2, P2Y4, and P2Y6 receptors on mature articular chondrocytes [11,38,39,40]. However, the present study provides the first evidence revealing the expression of additional P2Y12, P2Y13, and P2Y14 receptor subtypes in chondrocytes, as well as confirming the expression of P2Y1, P2Y2, P2Y4, and P2Y6. We also showed that the differentiation process highly upregulated the expression of *P2y1, P2y2,* and *P2y13* genes in ATDC5 cells. However, in contrast to *P2y2,* ADP-selective *P2y1* and *P2y13* appeared to be the two most abundant transcripts among all *P2y* studied. The expression of *P2y2* was formerly demonstrated to be significantly increased in differentiated ATDC5 cells relative to undifferentiated cells [31]. On the other hand, most purinergic receptors were detected throughout all the zones of articular cartilage, except for P2Y2 being more limited only to superficial zone cells [39].

Taking into consideration the fact that ADP and its stable analog ADPβS appeared to strongly modulate the migration of differentiated chondrocytes, one can presume that ADP-preferring P2Y receptors, namely P2Y1, P2Y12, or P2Y13 [10], might be involved in this process. Indeed, the changes in the activities of ADP and ADPβS are in accordance with the high abundance of *P2y1* and *P2y13* mRNAs in differentiated cells, implying that upregulated expression of those two GPCRs may partly be responsible for the control of cellular migration. Besides, the difference in *P2y1* and *P2y13* expression between differentiated and undifferentiated ATDC5 cells suggests the possibility that their ligands may be involved in the process of differentiation. Therefore, the next purpose of this study was to elucidate the influence of extracellular nucleotides on the differential/dedifferentiation characteristics of ATCD5 cells by investigating chondrogenic (*Col2a1*, *Sox9*), hypertrophic (*Runx2*), and fibrocartilaginous (*Col1a1*) mRNA expression. Differentiated ATDC5 cells specifically expressed the chondrogenic *Col2a1* transcript under the influence of ADP and ADPβS whereas undifferentiated cultures did not. ADP and ADPβS did not modulate the expression of the other genes under study. These data suggested that the highly elevated P2Y1 and P2Y13 receptors might be involved not only in the control of migration but also differentiation. This finding is consistent with the previous reports about mesenchymal stem cells (MSCs). It was shown that adipogenesis of MSCs is mediated through P2Y1 [41] whereas ADPβS stimulates MSCs’ differentiation into osteoblasts through P2Y13 [42].

The capability of ADP to enhance the migration and expression of the chondrogenic COL2A1 marker is relevant at the physiological level. The introduction of ADP or ADPβS into the extracellular space can potentially substantially improve the repair of cartilage. This hypothesis is consistent with a demonstrated role of platelet-derived ADP, but not ATP, as the key mediator for platelet-promoted chondrocyte proliferation and cartilage repair in osteoarthritis. Chondrocytes co-cultured with platelets showed a P2Y1-dependent increase in bone morphogenetic protein 7 synthesis, and transplantation of platelet-treated chondrocytes showed better cartilage repair in the OA model [43].

It should be noticed, however, that not all nucleotides can be considered as beneficial stimulators of chondrocyte physiology. Hereby, the ATP exposure of differentiated ATCD5 promoted a significant decrease in migration. ATP was previously shown to induce cell cycle arrest and suppress the proliferative and migration capacity of MSCs [44]. To date, multiple contradictory interpretations of the effects of ATP on chondrocytes have been reported. Potentially deleterious properties of ATP were described in the context of loss of the cartilage extracellular matrix. Bovine nasal cartilage resorption was promoted in the presence of ATP whereas ADP was inactive [45,46]. Extracellular ATP has, in contrast, a chondroprotective effect on bovine articular cartilage [46]. The exemplary opposite results show that the final effect exerted by extracellular ATP and other nucleotides is complicated due to a number of factors, including cell-specific expression of the receptor subtypes on the one hand and, on the other hand, ecto-nucleotidases and other enzymes responsible for nucleotide breakdown. These enzymes include members of ecto-NTPDases (ecto-nucleoside triphosphate diphosphohydrolases), ecto-NPPs (ecto-nucleotide pyrophosphatase/phosphodiesterase), ecto-alkaline phosphatases, and ecto-5′-nucleotidases [47]. The nucleotide kinases also participate in the regulation of the extracellular nucleotide concentration by transferring the phosphate moiety between nucleotides. Although little is known about linking nucleotides and ecto-nucleotidases with chondrogenic migration and differentiation, specific expression of ecto-nucleotidases members is responsible for regulating the extracellular concentrations of ATP, adenosine, inorganic phosphate, and pyrophosphate [48]. It should be assumed that the observations made during our research are not only the effect of nucleotide activity as P2Y receptor ligands but also enzymatical hydrolysis by ecto-nucleotidases that might be differentiation state-dependent expressed. Purinergic receptors and ecto-enzymes probably generate an orchestrated cellular signaling cascade essential for the development and maintenance of chondrocytes.

In summary, the results of this research demonstrate that the presence of extracellular nucleotides, especially ADP, ADPβS, and UDPβS, induces migration of differentiated ATDC5 cells. Additionally, ADP and ADPβS specifically induced expression of the chondrogenic *Col2a1* marker. This study also identified P2Y1 and P2Y13 receptors as possible regulators of the ADP- and ADPβS-stimulated chondrocyte migration and differentiation. We also intend to replicate the results on primary human chondrocytes to accurately and undoubtedly disclose the mechanisms and potential therapeutic value of extracellular nucleotides for cartilage repair. This novel finding both challenges our basic understanding of chondrocyte physiology and provides an opportunity for cartilage repair in the future. Our research is intended to supplement chondrocytes with exogenous nucleotides. A possible approach would be an incorporation of the most active compounds into scaffolds to stimulate cartilage regeneration. Since cartilage tissue engineering aims to obtain a structure mimicking native cartilage tissue through the combination of relevant cells, three-dimensional scaffolds, and extraneous signals, we think that incorporation of nucleotides into scaffolds could be used to successfully induce cartilage regeneration. The incorporation of different growth factors into various scaffolds has already been proven to stimulate chondrogenesis both in vivo and in vitro [49].

## 4. Materials and Methods

### 4.1. Tested Compounds

ADP, ATP, UDP, and UTP were purchased from Sigma-Aldrich (Merck KGaA, Darmstadt, Germany). Phosphorothioate analogs of nucleotides (TMPS, AMPS, UMPS, UDPβS, UTPγS, ADPβS, and ATPγS) were obtained from BioLog (Bremen, Germany).

### 4.2. ATDC5 Cell Culture

The chondrogenic mouse cell line ATDC5 was purchased from the Health Protection Agency (supplied by Sigma Aldrich, Saint Louis, MO, USA). Growth medium consisted of a mixture of Dulbecco’s modified Eagle medium/Nutrient Mixture F12 (DMEM/F12 1:1) supplemented with 2 mM glutamine (Thermo Fisher Scientific, Waltham, MA, USA), 5% fetal bovine serum (FBS, Thermo Fisher Scientific), and antibiotics (100 U/mL penicillin (Polfa Tarchomin, Warsaw, Poland), 100 mg/mL neomycin (Galfarm, Cracow, Poland), and 2.5 µg/mL amphotericin B (Thermo Fisher Scientific)). The culture was carried out at a temperature of 37 °C in a humidified atmosphere with 5% CO_2_.

### 4.3. Culture Media and Cell Differentiation

Two additional options (starving and differentiation medium) were used during experiments. Starving medium was composed of DMEM/F12 (1:1), 2 mM glutamine, and antibiotics. Differentiation medium was supplemented with Insulin-Transferrin-Sodium Selenite (ITS 100×, final compounds concentration in medium: 10 μg/mL insulin, 5.5 μg/mL transferrin, 67 ng/mL sodium selenite, Thermo Fisher Scientific), L-ascorbic acid (50 μg/mL, Sigma Aldrich), dexamethasone (100 nM, Sigma Aldrich), and Transforming Growth Factor – β2 (10 ng/mL, TGF-β2, Sigma Aldrich). Differentiated chondrocytes were obtained by a 14-day incubation in differentiation medium in 6-well plates (for RNA isolation) or 24-well plates (for migration research).

### 4.4. RNA Isolation and Reverse Transcription Quantitative Polymerase Chain Reaction

Extraction of the total RNA from ATDC5 cells was performed with a Universal RNA Purification kit (EurX, Gdańsk, Poland) according to the manufacturer’s recommendations. Subsequently, RNA was purified with DNase I (EurX) and then reverse-transcribed to cDNA with an NG dART RT kit (EurX). Primers for real-time PCR (Table 1) were designed with the NCBI’s Primer-BLAST software (National Center for Biotechnology Information; Bethesda, MD, USA) based on proteins’ sequences collected from the GenBank database and purchased from Genomed (Warsaw, Poland). Real-time RT-PCR was conducted using SG qPCR Master Mix (EurX) with a CFX96™ detection system (C1000 Touch, Bio-Rad, CA, USA). Real-time products were electrophoretically separated in 3% agarose gel in Tris/acetate/EDTA buffer enriched with nucleic acid stain (Midori Green Advance DNA stain, NIPPON Genetics, Japan). The expression of all genes was normalized to the expression of a Gapgh housekeeping gene. Relative gene expressions were calculated with the following formula:

(1) $$\mathrm{Relative}\;\mathrm{gene}\;\mathrm{expression}\;(1)=\;2^{-\Delta \mathrm{Ct}}$$

(2) ΔCt=[Ct(target genes ) - Ct(Gapdh)]

Increases of gene expression (in comparison to the control) were calculated with Livak’s method [24]:

(3) $$\mathrm{Increase}\;\mathrm{of}\;\mathrm{gene}\;\mathrm{expression}=\;2^{-\Delta \Delta \mathrm{Ct}}$$

(4) ΔΔCt=[ ΔCt(stimulated) - ΔCt (control)]

### 4.5. Cell Migration Studies

Confluent ATDC5 cell monolayers were used for the wound scratch migration test. A straight-edge scratch was made in each well with the use of a sterile 200-μL pipet tip. Wells were then rinsed with phosphate-buffered saline (PBS, Thermo Fisher Scientific), and starving or differentiation medium was added. Media were subsequently supplemented with tested compounds to a final concentration of 100 μM. Closure of the gap by cell migration was measured immediately (time 0) and 48 h after scratching with the Leica M205C microscope and Leica Application Suite software(Leica, Wetzlar, Germany). Cell migration was presented as a percentage of the control wound closure calculated as the formula:

(5) $$\mathrm{Reduction}\;\mathrm{of}\;\mathrm{scratch}=\;\frac{\Delta \;\mathrm{area}\;\mathrm{of}\;\mathrm{scratch}\;\mathrm{in}\;\mathrm{stimulated}\;\mathrm{cells}}{\;\Delta \;\mathrm{area}\;\mathrm{of}\;\mathrm{scratch}\;\mathrm{in}\;\mathrm{unstimulated}\;\mathrm{cells}}\times 100\%$$

where “∆ area of scratch in stimulated cells”—difference between scratch area at beginning of migration and after 48 hours of incubation in cells supplemented with tested compounds; and “∆ area of scratch in unstimulated cells”—average difference between scratch area at beginning of migration and after 48 hours of incubation in control cells.

### 4.6. Statistical Analysis

Cell migration and level of gene expression were analyzed by one-way ANOVA with a Bonferroni test. Results represent a mean value with a standard error of the mean. Two-way ANOVA was used to calculate the significance difference between the gene expression in differentiated and undifferentiated cells. Probabilities errors less than 0.05 were accepted as significant for all analyses. Differences between groups were rated significant at a probability error *p* < 0.05 (*), *p* < 0.01 (**), *p* < 0.001 (***), and *p* < 0.0001 (****). All calculations were performed with GraphPAD Prism 6 (GraphPad Software, La Jolla, CA, USA).

## Figures and Tables

**Table 1:** Sequence of primers used in RT-qPCR reaction.

Gene	NCBI Reference Sequence	Forward Primer	Reverse Primer
*P2y1*	NM_008772	TTATGTCAGCGTGCTGGTGT	CGTGTCTCCATTCTGCTTGA
*P2y2*	NM_008773	GAGGACTTCAAGTACGTGCT	ACGGAGCTGTAAGCCACAAA
*P2y4*	NM_020621	GGGGACAAGTATCGAAACCA	CACCCTCATAAGCAGGGAAG
*P2y6*	NM_183168	TGCTGTGTCAGAGGGAGTTTT	TCAGCCTTTCCTATGCTCGG
*P2y12*	NM_001357008	CTGAAGACCACCAGGCCATT	GGAATCCGTGCAAAGTGGAA
*P2y13*	NM_028808	GCACCAGAAGAGAGGCACAT	TAGGGGAAGAGTCGTCGTGT
*P2y14*	NM_001287124	GGAACACCCTGATCACAAAG	TGACCTTCCGTCTGACTCTT
*Col1a1*	NM_007742	ATGCCGCGACCTCAAGATG	TGAGGCACAGACGGCTGAGTA
*Col2a1*	NM_031163	AGGGCAACAGCAGGTTCACATAC	TGTCCACACCAAATTCCTGTTCA
*Runx2*	NM_009820	CACTGGCGGTGCAACAAGA	TTTCATAACAGCGGAGGCATTTC
*Sox9*	NM_011448	TCCCCGCAACAGATCTCCTA	AGGTGGAGTAGAGCCCTGAG
*Gapdh*	NM_008084	TCTCTGCTCCTCCCTGTTCC	CAATCTCCACTTTGCCACTGC

## Abbreviations

ACI	Autologous chondrocyte implantation
ADP	Adenosine 5′-O-diphosphate
ADPβS	Adenosine 5′-O-β-thiodiphosphate
AMPS	Adenosine 5′-O-thiomonophosphate
ATP	Adenosine 5′-O-triphosphate
ATPγS	Adenosine 5′-O-γ-thiotriphosphate
DMEM	Dulbecco’s modified Eagle medium
FBS	Fetal Bovine Serum
ITS	Insulin-Transferrin-Sodium Selenite
MSCs	Mesenchymal stem cells
OA	Osteoarthritis
PBS	Phosphate-buffered saline
RT-qPCR	Reverse transcription quantitative polymerase chain reaction
SEM	Standard error of the mean
TGF-β2	Transforming Growth Factor β2
TMPS	Thymidine 5′-O-thiomonophosphate
UDP	Uridine 5′-O-diphosphate
UDPβS	Uridine 5′-O-β-thiodiphosphate
UMPS	Uridine 5′-O-thiomonophosphate
UTP	Uridine 5′-O-triphosphate
UTPγS	Uridine 5′-O-γ-thiotriphosphate
